# PRE-OPERATIVE BILIARY DRAINAGE IN THE PERIAMPULLARY NEOPLASIA - A SYSTEMATIC REVIEW

**DOI:** 10.1590/0102-672020180001e1372

**Published:** 2018-07-02

**Authors:** Gustavo Costa Marques de LUCENA, Rinaldo Antunes BARROS

**Affiliations:** 1 Escola Bahiana de Medicina e Saúde Pública, Salvador, Bahia, Brazil

**Keywords:** Ampulla of Vater, Neoplasms, Drainage, Bile, Obstructive jaundice, Ampola hepatopancreática, Neoplasias, Drenagem, Bile, Icterícia obstrutiva.

## Abstract

***Introduction*:**

Periampular neoplasms represent 5% of all cancers of the gastrointestinal tract with peak incidence in the 7^th^ decade of life. The most common clinical picture is jaundice, weight loss and abdominal pain. Considering that cholestasis is related to postoperative complications, preoperative biliary drainage was developed to improve the postoperative morbidity and mortality of icteric patients with periampular neoplasias, whether resectable or not.

***Objective*:**

To describe the outcome of patients with periampullary tumors undergoing preoperative biliary drainage with pancreatoduodenectomy.

***Method*:**

The search was performed in the Medline/PubMed and Virtual Health Library databases by means of the combination of descriptors of the Medical Subject Headings. Inclusion criteria were clinical trials, cohorts, studies that analyze the morbidity and mortality of preoperative biliary drainage in Portuguese, English and Spanish. Exclusion criteria were studies published more than 10 years ago, experimental studies, systematic reviews and articles with WebQualis C or smaller journal in the area of ​​Medicine I or Medicine III. Of the 196 references found, 46 were obtained for reading with quality assessed through the Checklist Strengthening the Reporting of Observational Studies in Epidemiology. Eight studies were selected for review.

***Results*:**

A total of 1116 patients with a sample ranging from 48 to 280 patients and a mean age of 48 to 69 years were obtained. Of the eight studies, four observed a higher rate of bleeding in drained patients; three a higher rate of positive bile culture in the intervention group; site and cavitary infection, and biliopancreatic leaks were more common in the drainage group in two studies each. The death outcome and rate of reoperation were observed in larger numbers in the control group in one study each.

***Conclusion*:**

Preoperative intervention leads to a higher rate of infectious complications and bleeding.

## RESUMO

*****Introdução*** ****:**

Neoplasias periampulares representam 5% de todos os cânceres do trato gastrointestinal com pico de incidência na sétima década de vida. O quadro clínico mais comum é icterícia, perda de peso e dor abdominal. Considerando que a colestase está relacionada às complicações pós-cirúrgicas, a drenagem biliar pré-operatória foi desenvolvida objetivando melhorar a morbimortalidade pós-operatória de pacientes ictéricos com neoplasias periampulares, sejam elas ressecáveis ou não.

*****Objetivo*** ****:**

Descrever o desfecho de pacientes com tumores periampulares submetidos à pancreatoduodenectomia com drenagem biliar pré-operatória.

*****Método*** ****:**

A busca foi realizada nas bases de dados Medline/PubMed e Biblioteca Virtual em Saúde por meio da combinação de descritores do Medical Subject Headings. Os critérios de inclusão foram ensaios clínicos, coortes, estudos que analisam a morbimortalidade da drenagem biliar pré-operatória em português, inglês e espanhol. Os critérios de exclusão foram estudos publicados há mais de 10 anos, estudos experimentais, revisões sistemáticas e artigos com revista WebQualis C ou menor na área de Medicina I ou Medicina III. Das 196 referências encontradas, 46 foram obtidas para a leitura com qualidade avaliada através do checklist Strengthening the Reporting of Observational Studies in Epidemiology. Oito estudos foram selecionados para a revisão.

*****Resultados*** ****:**

Obteve-se 1116 pacientes com variação de amostra de 48 a 280 pacientes e média de idade de 48 a 69 anos. Dos oito estudos, quatro observaram maior taxa de sangramento em pacientes drenados; três maior taxa de cultura de bile positiva no grupo intervenção; infecção de sítio e cavitária, além de vazamentos biliopancreáticos foram mais comuns no grupo da drenagem em dois estudos cada. O desfecho morte e a taxa de reoperação foram observados em maior quantidade no grupo controle em um estudo cada.

*****Conclusão*** ****:**

A intervenção pré-operatória leva a maior taxa de complicações infecciosas e sangramentos.

## INTRODUCTION

Periampullary neoplasms represent 5% of all cancers of the gastrointestinal tract with peak incidence in the 7^th^ decade of life and affect more male patients than female patients (2:1)^6^ ^,^ ^19^ ^,^ ^29^. According to INCA, in 10 years, there were 313 cases per year^13^. They may appear relatively early due to obstruction of the biliary tract causing jaundice and pruritus that lead the patient to seek medical attention^6^ ^,^ ^19^. The most common symptoms are nonspecific, such as jaundice (present in 70-80% of patients), usually progressive and pruritus, weight loss and abdominal pain (present in 33%)^6^. However, periampullary neoplasms may also present with anorexia, generalized weakness, depression, iron deficiency anemia, nausea, pancreatitis, dyspeptic symptoms and elevation of hepatic enzymes^6^ ^,^ ^19^. The symptoms may vary according to their histological origin and cancers of pancreatic origin tend to be more aggressive^6^.

Tumor staging is a very important phase to decide the therapy, since it will be fundamental for the decision making about the technique used^29^; and, to that end, the Vienna and TNM classifications were proposed^4^ ^,^ ^25^.

The treatment of the resectable periampular tumor is a great surgical challenge due to difficulties in all phases, from diagnosis to the therapeutic process^18^ ^,^ ^29^. The treatments for them are well-established - Whipple procedure and endoscopic papilectomy^6,22,29^ -, but the former presents a considerable rate of morbidity (27-52%) and mortality (3-9%); and the second, despite the lower morbidity rate (19-33%) and mortality (0-3%), presented a high recurrence, reaching 35%; therefore, it is restricted to benign and small lesions (<2 cm)^1^ ^,^ ^19^.

Preoperative biliary drainage (PBD) has been developed since 1960 with the objective of improving the postoperative morbidity and mortality of icteric patients with periampullary neoplasms, whether resectable or not, with the objective of reducing postoperative morbidity and mortality and improving patients’ quality of life^20^ ^,^ ^28^. Obstructive jaundice and hyperbilirubinemia were identified as risk factors for peri and postoperative complications^27^. Preoperative biliary drainage can be performed by placing a stent, either by endoscopic retrograde cholangiopancreatography or by interventional radiology with a percutaneous approach^16^.

Preoperative biliary drainage is a topic discussed for decades and so far there is no concrete definition about its benefit or harm. Proponents of the procedure advocate that because of high levels of bilirubin indicate an increased risk of postoperative complications - in addition to liver dysfunction, impaired digestion, absorption of fat-soluble lipids and vitamins, coagulopathy, cholangitis, nephropathy, and in the late stages , hepatic insufficiency - PBD presents itself as a procedure capable of reducing these complications^16^. However, recently, studies have presented exactly the opposite results to the PBD, with similar or higher rates of morbidity, longer hospitalization time and higher costs^8^ ^,^ ^15^.

In view of the above, it is necessary to question the outcome of patients who undergo PBD. Thus, this systematic review is justified by the scientific gap of more information about the benefits or harms of this intervention. 

This study aims to describe the outcome of patients with periampullary tumors submitted to preoperative biliary drainage pancreatoduodenectomy.

## METHODS

Systematic review of literature with searches in electronic data sources Medline/PubMed and Virtual Health Library, performed through the combination of descriptors, including terms of the Medical Subject Headings (MeSH). It used publications in English, Portuguese and Spanish. The descriptors used for the search were related to the condition of the patient (obstructive jaundice OR jaundice) AND preoperative interventions performed (drainage OR stenting OR biliary stenting OR biliary drainage) AND surgical procedure (pancreaticoduodenectomy OR pancreatoduodenectomy OR duodenopancreatectomy). References in the articles identified by the search strategy were also searched manually to add to the study and literature review.

Inclusion criteria were randomized clinical trials, controlled clinical trials, clinical trials, retrospective cohorts, cohorts, and studies comparing preoperative biliary drainage with early operation.

Exclusion criteria were studies published more than 10 years ago, studies done on animals, systematic reviews, studies in which pancreaticoduodenectomy was done due to non-periampular tumors, journal articles with WebQualis C or less in the areas of Medicine I or Medicine III.

Each author, independently, read the titles and abstracts of each pre-selected paper in order to identify only the studies that correctly met the inclusion criteria. The articles were read separately by the authors in order to ensure the criteria for systematic review. Only articles that met 75% or more of the criteria of the Strengthening the Reporting of Observational Studies in Epidemiology (STROBE) checklist were selected.

Of the 196 references found through the search strategies, 46 were obtained for reading, when applied exclusion criteria related to time, language, study design and not obtaining in full the article. Of these, 42 articles were excluded because they did not address the periampullary neoplasia or compare results of preoperative biliary drainage with the early operation or did not address pancreatoduodenectomy; nine studies were excluded because they were case reports; another seven were excluded because they were systematic or literature reviews, and one because they were still in progress. At the end of the eligibility, three articles would meet the criteria proposed for the systematic review and another five were added by manual search selection of the references found.

## RESULTS

**FIGURE 1:**
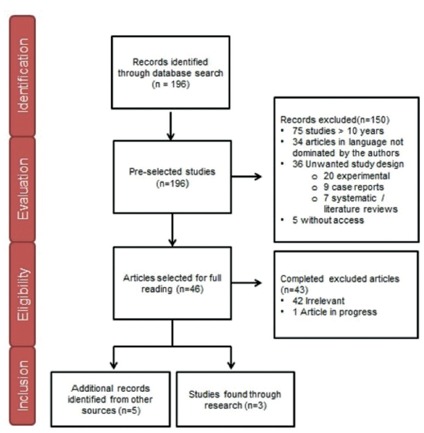
Flowchart of the selection process

Source: Lucena GCM & Barros RA, 2016

A total of 196 studies were selected, of which 193 were excluded, totaling three carefully selected articles, along with five other manual searches (Figure 1).

Samples ranged from 48 to 280 patients (n=1116 total) with mean age of 48-69 years (Table 1).

**TABLE 1:** Demographic profile of the studies obtained in the systematic review

Reference	Country, year	n	Mean age	Type of study
Bhati *et al.*	United Kingdom, 2007	48	48 years^1^; 50 years^2^	Retrospective cohort
Mezhir *et al.*	USA, 2009	188	69 years ^1^; 68 years ^2^	Prospective cohort
Herzog *et al.*	Germany, 2009	80	61 years ^1^; 66 years	Prospective cohort
Abdullah *et al*.	Singapore, 2009	82	62 years ^1^; 65 years ^2^	Retrospective cohort
Coates *et al.*	USA, 2009	90	65 years ^1^; 66 years ^2^	Retrospective cohort
Morris-Stiff *et al.*	United Kingdom, 2011	280	65,6 years ^1,2^	Prospective cohort
van der Gaag *et al.*	Netherlands, 2010	196	64,7 years ^1,2^	Randomized clinical trial
Arkadopoulos *et al.*	Greece, 2014	152	58 years ^1^; 57 years ^2^	Retrospective cohort

1: Control group; 2: Experimental group

Source: Lucena GCM & Barros RA, 2016

Periampullary neoplasia is a serious disease affecting mainly elderly patients, resulting in a clinical picture that decreases the quality of life with jaundice and pruritus, in addition to having a low life expectancy^6^. Preoperative biliary drainage is idealized as a method of reducing complications and mortality rates of pancreatoduodenectomy, believing that it provides better quality of life and death^6^.

The communication of the external environment with the biliary tract or the intervention itself seems to have favored the infection, as was found in the study by Bhati *et al*. and Mezhir *et al*. in 2007 and 2009, respectively, which showed a higher incidence of operative wound infection in the group that performed PBD^3^ ^,^ ^20^, with p=0.037 in the first study and p=0.01 in the second. These findings were compatible with those found by Garcea *et al*. and Sohn *et al*. in 2010 and 2000, respectively^9^ ^,^ ^26^, which identified an increase in the rate of surgical wound infection in patients submitted to PBD, similar to that found in the review made by Lai *et al*. in 2014 and by other authors^7^ ^,^ ^11^ ^,^ ^12^ ^,^ ^16^.

The studies of Mezhir *et al*. in 2009 and by Arkadopoulos *et al*. in 2014, found a positive relation between preoperative biliary drainage and subsequent formation of intra-abdominal abscesses with values of p=0.03 and p=0.02, respectively^2^ ^,^ ^20^. Similar findings were found by Cortes *et al*. in 2005 in a study with 79 individuals^7^.

Mezhir *et al*. (2009), Herzog *et al*. (2009) and Morris-Stiff *et al*. (2011), identified a positive relation between bile duct intervention (PBD) and the presence of bacteria in bile through culture, with statistical significance of p<0.001; p<0.001; and p=0.000002, respectively^10^ ^,^ ^20^ ^,^ ^21^. This fact is in agreement with what was found by other four authors of the world literature^9^ ^,^ ^17^ ^,^ ^23^ ^,^ ^24^.

As regards the sepsis complication, only Bhati *et al*. in 2007, among the articles of the results and the articles discussed, found a statistically significant difference between the control and intervention groups, with p=0.0183.

The presence of biliary leak was considered as a bile leakage greater than 50 ml, and this complication was observed more frequently in the group that performed the PBD only by Bhati *et al*. in 2007 (p=0.043)^3^, and this relation did not was found in none of the articles sought in the literature that evaluated this^9^ ^,^ ^11^ ^,^ ^12^ ^,^ ^14^. In 2011, Morris-Stiff *et al*. found a greater incidence of pancreatic extravasation - defined as a pancreatic leakage greater than 50 ml of liquid with amylase concentration three times higher than the upper limit - in the group that performed PBD, and this result was not found in others studies^3^ ^,^ ^7^ ^,^ ^11^ ^,^ ^17^ ^,^ ^18^ ^,^ ^21^ ^,^ ^23^.

Mezhir *et al*. (2009), Coates *et al.* (2009), Morris-Stiff *et al.* (2011), and Arkadopoulos *et al.* (2014), found a relation between preoperative biliary drainage and increase of bleeding (intraoperative or postoperative), a fact that was also found by Hodul *et al.* in 2003 in their retrospective cohort^2,5,11,20.23^. However, most of the studies found in this review pointed out that there is no statistically significant relation between preoperative biliary drainage and increase of bleeding^7^ ^,^ ^9^ ^,^ ^12^ ^,^ ^14^ ^,^ ^17^ ^,^ ^18^.

The need for reoperation was seen by Coates *et al*. in 2009, which identified a greater need for reoperation in patients who did not undergo preoperative biliary drainage (15% vs. 4%, p=0.02), going against what was identified by two other authors who evaluated this question, such as Martignoni *et al.*, in 2001, and Hodul *et al.*, in 2003, with a statistically non-significant result^5^ ^,^ ^11^ ^,^ ^18^.

Evaluating the total of complications, van der Gaag *et al.*, in 2010, Morris-Stiff *et al.*, in 2010, and Arkadopoulos *et al.*, in 2014^2^ ^,^ ^21^ ^,^ ^29^, found that preoperative biliary drainage is related to higher complication rates in patients who underwent (p<0.001, p=0.03, p=0.04, respectively); however, studies such as those of Huang *et al*. have identified that both groups of patients had similar rates of complications^8^ ^,^ ^12^. Despite this, Ngu *et al.*, in 2013, found a reduction in the rate of complications in patients who underwent DBPO (p<0.05)^23^.

As for another factor evaluated, length of hospital stay, it was seen that, in the study by Arkadopoulos *et al.*, in 2014, patients drained before the operation had a longer hospitalization period than those who went directly to the operation (11±6 vs. 16±8 days, p=0.0001)^2^. This finding partially corroborates what was identified by Huang *et al.*, in 2015, who identified that patients who perform endoscopic biliary tract drainage stay more time at the hospital^12^. And according to the study by Huang *et al.*, in 2015, patients who perform percutaneous transhepatic biliary drainage stay for less time in the hospital unit^12^.

Mezhir *et al.*, in 2009, did not identify an increase in the death rate in patients who did not perform preoperative biliary drainage, corroborating what had been observed by Hodul et al. (2003), Santos et al. (2003, 2005), Lermite et al. (2008) and Garcea et al. (2010)^7^ ^,^ ^9^ ^,^ ^11^ ^,^ ^12^ ^,^ ^17^ ^,^ ^18^ ^,^ ^23^ ^,^ ^24^. Mortality was evaluated in-hospital, before 30 days after the intervention, on the 30^th^ and 90^th^ day after the operation.

The studies discussed in this section with their respective year of publication, location, n, main results and limitations, can be identified in Table 2.

**TABLE 2:** Main results and respective limitations of studies found

Author, year of publication	Study site	n	Main results	Limitations
Bhati CS et al, 2007	United Kingdom	48	Sepsis (p »0.018). operative wound infection(p = 0.037) and small bile leakage(p = 0.043)were higher in the PBD group than in the control group	Then of the study is small; part of the patients were not drained in the center that carried out the study
Mezhir JJ et al. 2009	USA	188	Surgical wound infection (p = 0.01). infections (p = 0.002), intra-abdominal abscess (p =0.03). mean intraoperative blood loss(p = 0.04), and positive bile culture (p <0.01) were higher in the PBD group. However, the death outcome was more present in the non PBD group.	
Herzog T e tal. 2009	Germany	80	The DBPO group presented a higher percentage of positive bile culture intraoperatively (p <0.001).	Then of the study is small
Abdullah SA et al. 2009	Singapore	82	The rate of surgical wound infection of the control group was higher than the intervention group(p = 0.01)	Then of the study is small
Coates JM et al. 2009	USA	90	The PBD group presented greater dissemination to regional lymph nodes (p =0.001) and greater blood loss (p =0.03). The control group had a higher reoperation rate (p = 0.02).	Then of the study is small
Morris-Stiff G et at, 2001	United Kingdom	280	The PBD group had a higher complication rate (p = 0.03), higher number of positive cultures (p = 0.000002), more pancreatic leakage (p =0.013) and gastrointestinal or intraabdominal bleeds (p = 0, 03)	
van der Gaag NA et al., 2010	Netherlands	196	The patients in the control group had a lower rate of complications than the intervention group(p <0.001).	The groups were not equivalent, the DBPO group had more men (p = 0.01) and was leaner (p = 0.03) than the control group
Arkadopoulus et al. 2014	Greece	152	The control group had shorter surgical time(p <0.00001), lower intraoperative blood loss (p = 0.0016). and lower number of committed lymph nodes (p = 0.0077). The intervention group had more infected intra-abdominal collections (p = 0.02), chest infection (p =0.03). morbidities percentage (p =0.04) and hospitalization time (p = 0.0001).	

Source: Lucena GCM & Barros RA, 2016.

This systematic review has limitations since only half of the studies analyzed were prospectively designed and only one was a multicenter randomized clinical trial. Another limitation is a relatively small n if we add up the number of samples from all studies. Thus, a larger number of clinical trials with a greater number of patients are needed to elucidate the true role of preoperative biliary drainage in periampullary neoplasia, addressing both the way (percutaneous or endoscopic) and its main mortality.

However, this review used strict criteria for the selection of the articles to be analyzed in order to reduce bias risks and to guarantee the quality of each of the studies found. In addition to this, this paper stands out for the pioneering methodology applied as a systematic review, and this study is the first in the Portuguese language besides representing the existence of few as a whole in the approach to this theme.

## CONCLUSION

The main outcomes of patients with periampullary neoplasia and submitted to preoperative biliary drainage were infection, positive bile culture, surgical wound infection and formation of intra-abdominal abscesses and bleeding, without generating a longer hospitalization time.
